# Lady Macbeth's Night Walking With Dissociative Symptoms Diagnosed by the First Sleep Medicine Record

**DOI:** 10.3389/fpsyg.2020.563773

**Published:** 2021-01-25

**Authors:** Marleide da Mota Gomes, Antonio E. Nardi

**Affiliations:** ^1^Laboratory of History of Psychiatry, Neurology, and Mental Health, Institute of Psychiatry, Institute of Neurology, Federal University of Rio de Janeiro, Rio de Janeiro, Brazil; ^2^Laboratory of History of Psychiatry, Neurology, and Mental Health, Institute of Psychiatry, Federal University of Rio de Janeiro, Brazilian Academy of Science, National Academy of Medicine, Rio de Janeiro, Brazil

**Keywords:** sleep, insomnia, depression, sleepwalking, dissociative disorder, conversion disorder

## Introduction

William Shakespeare was an innovative play writer prone to capture in his works the knowledge of his time, at the English Renaissance that includes the Elizabethan and early Jacobean Eras (Gomes, 2015). Much of Shakespeare's work was assembled at The First Folio (Shakespeare, 1623) that is Bard's 35 plays post-mortem collection that is considered one of the most influential books ever published. Lady Macbeth is one of the main characters of the Macbeth Tragedy, wife of the protagonist whom she convinced to kill King Duncan, but she ends up severely plagued by her guilt (Shakespeare, 1988).

Many texts of Shakespeare may be considered a lesson of psychopathology (Levin, 2002). This is very interesting when it is considered that very little was known in Shakespeare's day about mental disorders as to somnambulism/night walking. The most detailed data could be read in some plays as in The Tragedy of Macbeth (Shakespeare, 1988). Night walking could be interpreted at this period as a prophetic or spiritual moment in which the individual was believed to be under the influence of strange or paranormal forces (Kocher, 1954; Maraldi, 2019). For some it was considered a prophetic or ecstatic state, but for others it was the result of a badly conducted baptism ceremony, consequently, they were often called the ill-baptized (Munro, 1887).

Sleep disorders are among the most expressive of Shakespeare's work, regarding medical issues (Riva et al., 2010). Probable, it reflects some publications on sleep problems and dreams (Riva et al., 2010) that were common in daily discussion in Jacobean England, and night walking was defined as a “serious disorder, a form of melancholy” (Riva et al., 2010), and “a great agitation of the brain” (Riva et al., 2010). Regarding the sleep problems in general, they were found in several plays, but undoubtedly, the most famous is found in The Tragedy of Macbeth (Act 5, Scene 1/Act 2, Scene 2/Act 3, Scene 2/Act 5, Scene 3) (Shakespeare, 1623).

An initial survey made on an edition of the First Folio shows that the word sleep appears 329 times, besides fall which appears 337 times, it is the most found among others that may be related to the neuropsychiatric area, surpassing fit (221 times) and faint (52 times) (Shakespeare, 1623).

The play about Macbeth was probably written for King James's ancestors and the Stuarts' accession to the English throne in 1603. The King demonstrated curiosity in night walking because he and his mother, “Mary, Queen of Scots, suffered from fits with loss of consciousness and sleep complaints” (Riva et al., 2010).

This paper discusses Lady Macbeth's night walking on Shakespeare's report, mainly regarding the relationship between a non-rapid-eye-movement sleep (NREM) and REM parasomnia, and dissociative disorders (DD).

## Lady Macbeth's Nightly Ambulatory Behavior

Regarding Bard's works, Shakespeare deeply and unashamed scrutinizes the human behavior and feelings what is masterfully presented employing a scene of a vital physiological phenomenon for the human being that was disrupted. At the Tragedy of Macbeth, several of its characters suffer from sleep problems, usually associated with guilt and punishment, feelings for their sins. This punishment is intense and painfully presented in a kind of sleep laboratory, as it was shown in Lady Macbeth, Act 5, scene 1 (Shakespeare, 1623). It can be observed that Lady Macbeth take part as the subject of a first recorded sleep laboratory scene, as her doctor examine her behavior during the night, at the side of the gentlewomen. Act 5, scene 1 of a night walking suggests a vivid NREM-parasomnia or even a DD, all of them probably present in this case. However, firstly we will present some issues regarding sleep phases and eventual intrusion in wakefulness.

Abnormal nocturnal behaviors exhibit various symptoms from simple motor activities to complex and severe behaviors. The illnesses of nocturnal abnormal behaviors include parasomnias, epilepsy, and mental disorders (American Psychiatric Association, 2013).

The parasomnia may be those of NREM-related parasomnia, REM-related parasomnia, and other parasomnias according to the International Classification of Sleep Disorders-3 (ICSD-3) (Singh et al., 2018).

NREM-related parasomnia is classified as Confusional arousals, Sleepwalking (SW), Sleep terrors, and Sleep-related eating disorder; and REM-related parasomnia are subdivided into REM sleep behavior disorder, Recurrent isolated sleep paralysis, and Nightmare disorder according to ICSD-3 (Singh et al., 2018).

Parasomnias are more often seen in children than in the adult population, however, REM Sleep Behavior Disorder can be associated with various neurologic disorders like α-synucleinopathies, Parkinson's disease, and narcolepsy (Singh et al., 2018).

**Figure 1:**
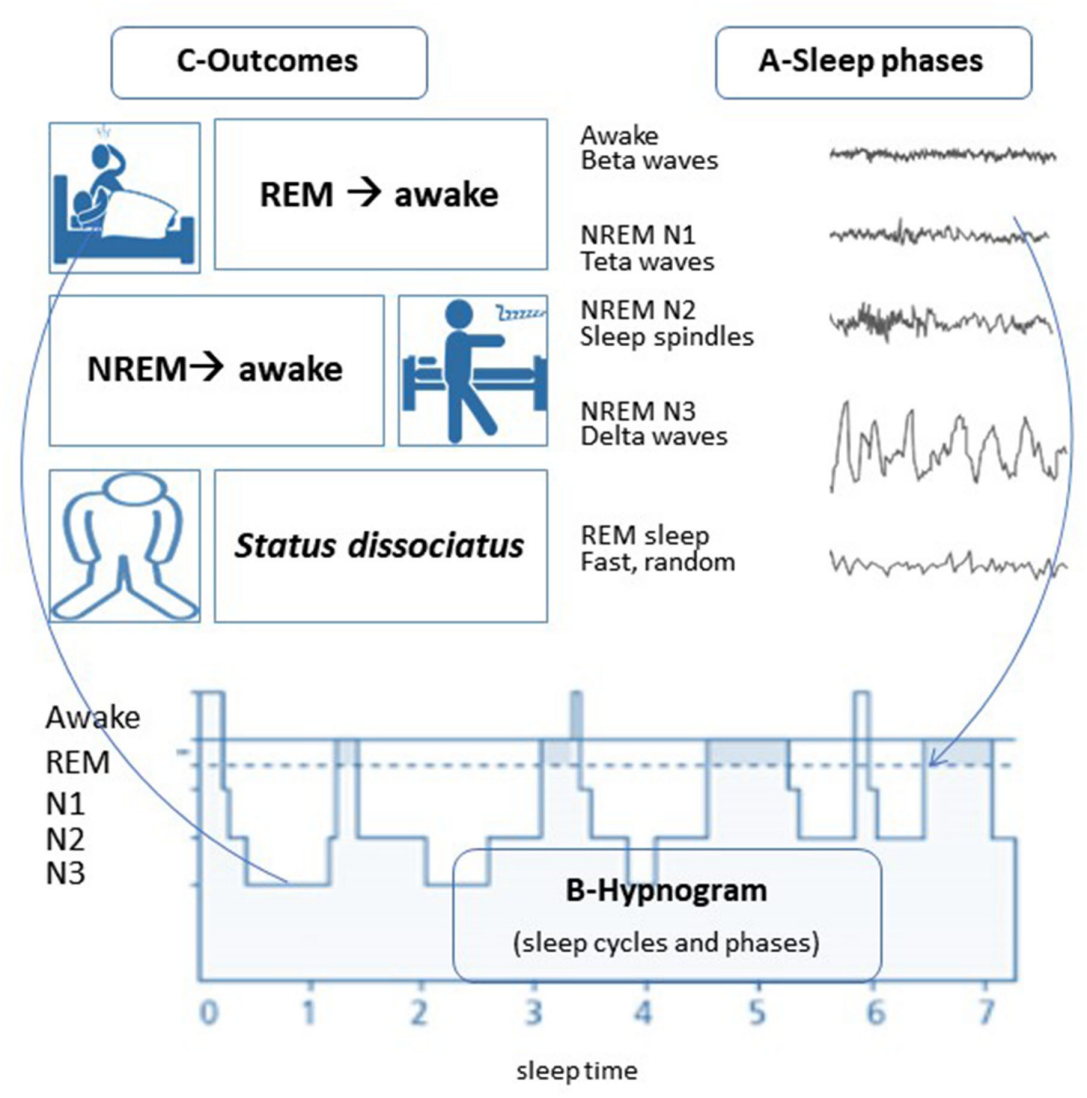
The sleep phases and their intrusions in wakefulness. **(A)** Sleep phases and their characteristics in EEG, REM, and NREM sleep; **(B)** Hypnogram (summary of basic sleep characteristics along time) showing sleep cycles, each with sleep phases (NREM-N1, N2, N3, and REM); C- Intrusions of the sleep phases into wakefulness and their results. Illustration by M. da Mota Gomes.

This SW disorder can be one of the parasomnias that are a group with abnormal and unpleasant verbal or behavioral motor signs that may appear during sleep or wake-up to sleep transitions (Figure 1).

Each sleep phase has its characteristics, and more commonly vivid dreams occur at the REM sleep phase when there is body paralysis, and fortunately, dream enactment is not possible. However, abnormal patterns may occur due to the intrusion of sleep phases into wakefulness or vice-versa. REM sleep intrusion in the waking state may also explain cataplexy, sleep paralysis, and hypnagogic (wake to sleep) or hypnopompic (sleep to wake) hallucinations, as is mostly seen in narcoleptic patients.

Disrupted sleep may also suppress, at least temporarily the motor-suppressive activity in REM sleep, and consequently promotes REM behavior disorder. In this situation, there is a process of dream enactment that likely begins with active, often emotionally charged dream content that may occasionally break through the normal REM sleep motor suppressive activity. This behavior may be expressed as kicking, punching, yelling, jumping, or aggressive behavior associated to a dream (Singh et al., 2018).

The intrusion of REM sleep in wakefulness may also occur at the post-traumatic stress disorder, and other sleep disorders, that share the possibility of body enact dream content and it underlies oneiric experience that can be expressed by dream enactment behavior. Many recent works shed light on this question (Baltzan et al., 2020; Barone, 2020; Elliott et al., 2020; Rocha and Arnulf, 2020; Siclari et al., 2020).

Posttraumatic stress disorder increases the chances for REM sleep behavior disorder and other parasomnias in people with and without comorbid traumatic brain injury, as in the veterans how is demonstrated in the study by Elliott et al. (2020).

In contrast to REM sleep behavior disorder, NREM parasomnias have often been related as non-dreaming states, as they may appear from N3 sleep that is one sleep stage with minimal dream recall. However, some data from sleep laboratories show that dreams may also be enacted in NREM parasomnia (Rocha and Arnulf, 2020; Siclari et al., 2020).

Regarding the confusional arousals, they are episodes of confusion, disorientation, and bizarre behavior immediately after awakening from sleep. As to the night terrors, there is a terrifying scream with severe autonomic symptoms and often motor activity, which might be stereotyped (Hartman et al., 2001).

Precisely, SW is a deambulatory movements with decreased levels of consciousness, which may occur during incomplete arousals mainly from slow-wave sleep. Patients act semi- purposefully and usually have poor memory of related events that are less unusual and original than that of REM dreams.

However, may occur an overlap between non-REM parasomnia and REM sleep behavior disorder that is associated with a history of dream-enacting with dream recall. This overlapping represents an apparent boundary confusion between stages of non-REM and REM sleep. Besides, it may occur a rare and severe dissociation between non-REM, REM, and wake states what constitutes *agrypnia excitata* or *status dissociatus*, resultant clinically in oneiric behaviors and severe disorganization of normal polysomnographic wake and sleep stage features (Hrozanova et al., 2019). Regarding *status dissociatus*, it can happen in alcohol withdrawal, autoimmune encephalitis, or in synuclein neurodegenerative disorders (Hrozanova et al., 2019).

The extreme form of *status dissociatus, agrypnia excitata*, may occur in alcohol withdrawal, Morvan syndrome, or fatal familial insomnia and other prion diseases (Hrozanova et al., 2019).

There are commonly reported trigger factors for SW/non-REM parasomnia that include sleep deprivation and stress (Bargiotas et al., 2017), as happened with Lady Macbeth. SW is usual in school children and rare in adults, and it may be trigger by genetic features, neurological, psychiatric factors, but its diagnosis is based on anamnesis, and video-polysomnography may be needed to exclude some similar disorders. Indeed, the identification of SW episodes is deeply reliant on the behavior being detected by others or the sleepwalker questioning it because of injuries or other manifestations when he/she awake. Treatment of SW needs the recognition of etiological factors, means for the sleep environment to become safer, and the use of sedative drugs, if necessary.

“Gentlewoman: Since his majesty went into the field, I have seen her rise from her bed, throw her night-gown upon her, unlock her closet, take forth paper, fold it, write upon't, read it, afterwards seal it, and again return to bed; yet all this while in a most fast sleep.”

“Gentlewoman: Neither to you nor any one; having no witness to confirm my speech. Enter LADY MACBETH, with a taper. Lo you, here she comes! This is her very guise; and, upon my life, fast asleep. Observe her; stand close.”

“Doctor: You see, her eyes are open. Gentlewoman: Ay, but their sense is shut.” “LADY MACBETH: Wash your hands, put on your nightgown; look not so pale. …I tell you yet again, Banquo's buried; he cannot come out on's grave.” (Shakespeare, 1623)

When Lady MacBeth's physician gave his impression about her sleep problem to his husband, Macbeth recognized the psychogenic aspects underneath the night walking and the possible way to treat it.

Indeed, the NREM-related parasomnias have many differential diagnoses, including REM sleep behavior disorder, sleep-related epilepsy, sleep-related DD, alcohol-and drug-related behavioral signs and symptoms during sleep, obstructive sleep apnea, and psychogenic events or malingering (Hartman et al., 2001). Naturally, a significant psychosocial antecedent, as occurred with Lady Macbeth raises a possible diagnosis of a DD and post-traumatic stress disorder with dream enactment behavior.

The dissociative experiences, such as those occurring in DD, may also be comorbid with episodes of SW, but occurring in some moments during which the individual remains awake (Hartman et al., 2001). In the Statistical Manual of Diagnosis of Mental Disorders−5th Edition (American Psychiatric Association, 2013), a dissociative symptom is related to the deterioration of the consciousness, such as recalls of memory, self, and local orientation.

Karatas et al. (2017), determined that a portion of the patients with parasomnia also had dissociative disorders, with dissociative amnesia at 33.3%, dissociative evasion at 13.3%, and dissociative disorder that cannot be otherwise named at 53.4%, there was also sexual or physical abuse and neglect.

One more association between dissociative symptoms and sleep is raised when it is known that sleep hygiene may be very useful to the treatment or prevention of dissociative symptoms (Maraldi, 2019). It is a diagnostic challenge, as the principal differential diagnosis of Lady Macbeth's is between a vivid SW and a DD, not exclusively.

Lady Macbeth's scene portrays deambulatory behavior, SW that may suggest somnambulism, plus a dissociative disorder or even *dissociatus* status less probably post-traumatic stress disorder with dream enactment behavior.

## Discussion

Lady Macbeth presents a nocturnal dissociative episode with an altered state of consciousness, in which traumatic memories return, as a possible manifestation of post-traumatic stress disorder. However, in this case, the main question raised is whether we are facing the diagnosis of DD itself or a case of NREM-related parasomnias, particularly a vivid SW, combined with dissociative symptoms. The main trick point to this differential study is based on the level of consciousness. This favored our choice for the first option, vivid SW, as the Gentlewoman says: “Gentlewoman: Ay, but their sense is shut” that we can assume that the patient is asleep, and if she were awake, this would be a case of DD.

However, the vivid, complicated, lengthy night-walking in adults is not common in patients with SW. However, psychiatric comorbidities are found in patients with SW, mainly depression, as shown in 25% of the adult-onset SW by Bargiotas et al. (2017). Besides, commonly informed aetiological factors for SW comprise sleep deprivation and anxiety (Bargiotas et al., 2017).

Regarding Lady Macbeth, she had a paradox between her awake state of indomitable strong power, at the side of the sleeping one. This side was pressed by the shadows and the suggestions of the night favored by the liberty of conscience what yield and throw off forever the mask of the right person that she had worn so long (Munro, 1887). Regarding the dissociative disorder, they are frequent in the general population (Maraldi, 2019), with about one-third of individuals reporting at least one dissociative symptom during their stressful moments. The etiology of dissociation has been psychologically linked to childhood trauma, but even the trauma feature points to the need of considering cognitive, psychopathological, and sociocultural features, other than the trauma itself in a comprehensive model of dissociative symptoms (Maraldi, 2019). Besides, there is an increasing amount of data lightening the complex association between dissociation and anomalous sleep disorders such as recurrent nightmares, vivid and bizarre dreams, sleep paralysis, hypnagogic/hypnopompic imagery, and narcolepsy symptoms (Maraldi, 2019).

Concerning just the dream, it has been considered an interesting feature for dissociative disorders such as absorption/ imaginative involvement and multiple identity states (Crisp, 1996). Besides, it is frequent to consider the concrete and psychological theme of a person's dreams as a predictor of dissociative tendencies and conversion symptoms (Levin, 2002; Maraldi, 2019). Also, highly dissociative patients usually describe more vivid dreams and other unusual sleep experiences. However, Lady Macbeth's SW behavior is not favorable to REM sleep disorder and eventually linked dream enactment behavior as eyes open usually are not found in this condition as mentioned in the scene: “Doctor: You see, her eyes are open.” Besides the eyes usually closed, in REM sleep disorder, the patients are not aware of their environment, and they can't get far from their bed as Lady Macbeth did.

About 16–25% of adult sleepwalkers have comorbidity with depressive or bipolar disorders, as mentioned by Hrozanova et al. (2019), as seems to be the case of Lady Macbeth, which led her to suicide. Anyway, it is worth to recognize that dissociation disorder is a potential differential diagnosis for NREM parasomnias. This discrimination is a challenge and makes this an area of complex study. However, we can hypothesize that Lady Macbeth suffered from dissociative amnesia (AD) as well as depression (American Psychiatric Association, 2013). This disorder involves the temporary loss of recall memory which can be voluntary or involuntary and can result from stressful moments, with severe and disturbing emotions such as those that affected Lady Macbeth.

The close association between sleep disorders and dissociation is a poor explored subject that could open many hypothesis or etiological factors to dissociative syndromes from childhood trauma and to feelings (Kocher, 1954). Also, sleep disorders such as sleep paralysis and bizarre dreams have given rise to various interpretations from popular and paranormal beliefs, such as stories of alien abductions and the presence of strange and feared creatures during the night (Kocher, 1954; Maraldi, 2019). Indeed, SW may be an interesting tool for understanding the differences and similarities among beliefs about sleep-related phenomena in different cultures (Kocher, 1954; Hartman et al., 2001).

“MACBETH: Cure her of that. Canst thou not minister to a mind diseased, Pluck from the memory a rooted sorrow, Raze out the written troubles of the brain And with some sweet oblivious antidote Cleanse the stuff'd bosom of that perilous stuff Which weighs upon the heart? (Act 5, Scene 3)“ (Shakespeare, 1623)

**Figure 2:**
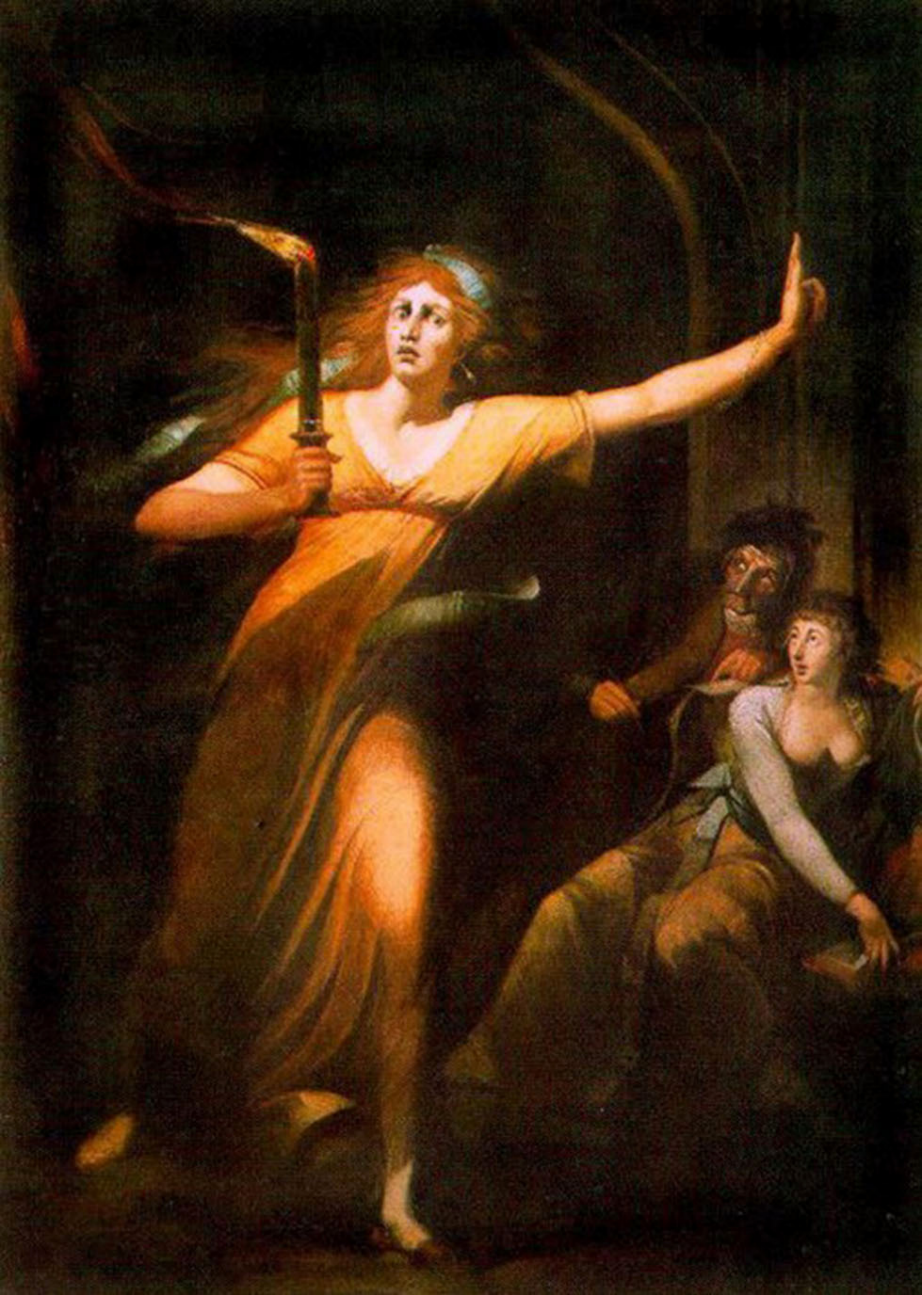
The Sleepwalking of Lady Macbeth by Johann Heinrich Füssli (1781–1784) (Musée du Louvre). Free domain.

Regarding the memory of the events, sleepwalkers usually have poor memory of SW events that are less unusual and original than REM dreams. Naturally, the self-reports and clinical observations have their bias on at least partial awareness of the event by the individual or being told about his/her SW by someone who has observed it, as the physician of Lady Macbeth. The use of light by Lady Macbeth (Figure 2) could also be considered another dissociative sign for a careful psychopathologist. However, the relationship between the mentation and full-blown episodes of SW and night terror may be higher than is currently thought. Some observations may point that dissociation is not explicitly associate with similar sleep disorders, but to the quality of sleep and insomnia symptoms, as we observe in Lady Macbeth. Besides, dissociation was also shown to be associated with a series of non-pathological anomalous sleep experiences, such as lucid dreaming and out-of-body experiences (Maraldi, 2019). Also, dissociative experiences seem to be associated with nightmares and waking dreams, but it is not correlated with “concious dreaming,” thereby suggesting that dissociation may be related to those sleep disturbances that are hard to manage.

However, the dissociation prevalence during anomalous sleep experiences is mostly unknown, either for general or clinical populations (Maraldi, 2019), but Hartman et al. (2001) considers a great association between parasomnia and dissociative disorders. Lady Macbeth's physician and his assistant, the Gentlewoman, used the clinical observation and detailed anamnesis, in this way, we could consider this scene as the first laboratory session described in the literature (Kocher, 1954). Regarding Lady Macbeth's experience, it is full of guilty and severe depression that leads her to sleep disturbance, and in the end, the character commits suicide. The underlying mood disturbances may have worked as a trigger for the SW and a final resolution of a sublime ambitious character. However, we can assume that we are not dealing with a simple non-REM parasomnia, but it can be a more complex one due to the imbrication of different phases of sleep as occurs in the *dissociatus* state. Besides, this non-REM / REM parasomnia can be bi-directionally related to dissociative phenomena (Crisp, 1996).

In short, William Shakespeare was a playwright who innovated the theater and analyzed human behavior in a creative, seductive, and shameless way. The Bard addressed medical issues steeped in human emotions, including the sleep disorders of his tormented characters. In the play, The Tragedy of Macbeth, the night walk of Lady Macbeth is analyzed through the words of her doctor and Gentlewoman. This scene can be considered the first record of sleep medicine described in the literature. The identification of episodes of night walks depends deeply on the behavior witnessed by other people. The diagnostic option regarding this event concerns a parasomnia associated with dissociative symptoms. Thus, it is possible to suggest that the traumatized Lady Macbeth had a *dissociatus* status resulting from the overlap of non-REM / REM sleep parasomnia and dissociative symptoms. These due to her anguish and/or her deep disturbing silent feelings.

## Author Contributions

Both authors planned the project together, read the references, discussed and planned the article, wrote the manuscript, agreed, and approved with its final version.

## Conflict of Interest

The authors declare that the research was conducted in the absence of any commercial or financial relationships that could be construed as a potential conflict of interest.
